# The Complex Landscape of Updated Pneumococcal Conjugate Vaccines

**DOI:** 10.1093/ofid/ofaf050

**Published:** 2025-01-28

**Authors:** Deus Thindwa, Eugene D Shapiro, Daniel M Weinberger

**Affiliations:** Department of Epidemiology of Microbial Diseases and the Public Health Modeling Unit, Yale School of Public Health, New Haven, Connecticut, USA; Department of Pediatrics, Yale School of Medicine, New Haven, Connecticut, USA; Department of Epidemiology of Microbial Diseases, Yale School of Public Health, New Haven, Connecticut, USA; Department of Epidemiology of Microbial Diseases and the Public Health Modeling Unit, Yale School of Public Health, New Haven, Connecticut, USA

## Abstract

Pneumococcus is a major cause of serious infections, especially among vulnerable populations. While pneumococcal conjugate vaccines (PCVs) provide effective protection against disease caused by the included serotypes, a substantial burden of disease remains. Several new PCVs are under development or were recently recommended for use to counteract the remaining disease burden. This had led to complicated policy deliberations on their optimal use in different populations. We discuss how key factors should be considered in any policy decision: serotype coverage of a new PCV, prevalence of the untargeted remaining serotypes, strength of the immune response to the serotypes in a new PCV, potential for PCV evasion, PCV costs, and optimal simultaneous use of PCVs in children and adults. We also suggest the need for robust analyses of available surveillance data and continual monitoring of changes in the pneumococcal serotypes that are responsible for disease and colonization to help decision makers make optimal recommendations.

*Streptococcus pneumoniae* (pneumococcus) is a major cause of respiratory infections, meningitis, and bacteremia, particularly among children <5 years old, older adults, and individuals who are immunocompromised [1]. While pneumococcal conjugate vaccines (PCVs) provide effective protection against disease caused by the targeted serotypes, a substantial burden of disease remains [2]. Originally consisting of 7 serotypes, approved PCVs now comprise up to 21 serotypes, and PCVs that include even more serotypes are under development [2]. PCV7 and PCV13 were introduced for children in 2000 and 2010, respectively, and PCV13 has been recommended for use in adults for over a decade [3]. By 2019, the incidence of invasive pneumococcal disease (IPD) due to serotypes in PCV13 and overall fell by 98% and 92% in children aged <5 years and 87% and 62% in adults aged ≥65 years, respectively [2]. In adults, there was a substantial increase in IPD due to nonvaccine serotypes, a phenomenon known as “serotype replacement” [4]. Recently approved PCVs take different approaches to expand protection [5], and there are trade-offs in terms of serotypes included, effects on serotype replacement, immunogenicity, and cost.

PCVs are composed of purified serotype-specific surface polysaccharides that are linked (conjugated) to a protein [6]. Vaccinated individuals generate an antibody response that is serotype specific [7]. PCVs are highly effective at preventing IPD and protect against pneumonia. Importantly, PCVs also reduce nasopharyngeal colonization with targeted serotypes. Young children are the main source of transmission of pneumococcus, so when children are vaccinated with PCVs, transmission in the population decreases [8]. Thus, vaccination of children affects disease burden in all age groups, including unvaccinated adults, and alters which serotypes are transmitted in the population [8, 9].

Increasing the number of serotypes in PCVs tends to decrease their immunogenicity [7], the consequences of which are not yet clear. It is likely that the immunogenicity of the new PCVs will still be sufficient to provide robust protection against IPD. However, higher antibody concentrations might be required to protect against mucosal infections (eg, pneumonia, otitis media) and/or prevent nasopharyngeal colonization. If protection against colonization is compromised, then indirect protection of unvaccinated individuals might not be maintained. It will be important to assess the magnitude of increased colonization with specific serotypes and the minimum concentration of antibodies needed to sustain indirect protection. Data in healthy children would likely provide the earliest indication of changes in rates of serotype-specific colonization, including possible reemergence of PCV-targeted serotypes following the switch to new higher-valent PCVs.

PCV15 and PCV20 were recently licensed for use in young children and older adults [10]. These PCVs add serotypes to those that are in PCV13. Other new pneumococcal vaccines under development (eg, Vax24, Vax31, and MAPS24) largely follow this same approach of increasing the number of serotypes included, though they use different technologies, which might affect their immunogenicity (Table 1) [11, 12]. These vaccines are all intended to be used in children and adults. In contrast, a recently licensed 21-valent vaccine (V116) takes a different approach to increase protection of adults against different serotypes [13]. The rationale for V116 is that adults are indirectly protected against many serotypes when uptake of PCVs in children is high, so vaccinating adults with the same vaccine might have limited benefit [14]. V116, intended for use only in adults, does not include many serotypes in PCV13, PCV15, or PCV20 but does include other serotypes that are important causes of disease in adults [13]. In low- and middle-income countries, the focus has been on reducing the costs of PCVs. Pneumosil, a lower-cost 10-valent vaccine, has fewer serotypes than other PCVs but focuses on the serotypes that cause the greatest burden of disease in low- and middle-income countries (Table 1) [15].

**Table 1.** Pneumococcal serotypes targeted by various pneumococcal conjugate vaccines (PCVs), including vaccines still under clinical investigations, and the proportion of invasive pneumococcal disease (IPD) preventable by each PCV.

	Pneumococcal Serotype																																			PCV Coverage^a^	
PCV	4	6B	9V	14	18C	19F	23F	1	5	7F	3	6A	19A	22F	33F	8	10A	11A	12F	15B	2	9N	17F	20B	15A	16F	20A	23A	23B	24F	31	35B	15C	7C	6C	<5 years (%)	50+ years (%)
PCV7	x	x	x	x	x	x	x																													11.8	9.1
PCV10 (GSK)	x	x	x	x	x	x	x	x	x	x																										11.8	9.4
PCV13	x	x	x	x	x	x	x	x	x	x	x	x	x																							28.6	27.5
PCV15	x	x	x	x	x	x	x	x	x	x	x	x	x	x	x																					43.7	39.4
PCV20	x	x	x	x	x	x	x	x	x	x	x	x	x	x	x	x	x	x	x	x																53.8	50.6
SP0202 (21-valent)	x	x	x	x	x	x	x	x	x	x	x	x	x	x	x	x	x	x	x	x		x														53.8	57.4
MAPS24	x	x	x	x	x	x	x	x	x	x	x	x	x	x	x	x	x	x	x	x	x	x	x	x												54.6	63.3
VAX24	x	x	x	x	x	x	x	x	x	x	x	x	x	x	x	x	x	x	x	x	x	x	x	x												54.6	63.3
PCV25 (Inventprise)	x	x	x	x	x	x	x	x	x	x	x	x	x	x	x	x	x		x	x		x			x	x				x		x			x	77.3	73.1
VAX31	x	x	x	x	x	x	x	x	x	x	x	x	x	x	x	x	x	x	x	x	x	x	x	x	x	x		x	x		x	x		x		79.8	90.9
Pneumosil^®^ (10-valent)		x	x	x		x	x	x	x	x		x	x																							14.3	6.3
V116										x	x	x	x	x	x	x	x	x	x			x	x		x	x	x	x	x	x	x	x	x			^b^	81.4

^a^PCV coverage – proportion of serotyped invasive pneumococcal disease (IPD) targeted by each PCV based on 2022 IPD data from the United States Centers for Disease Control and Prevention Active Bacterial Core (ABC) surveillance project. PCV coverage data were obtained from https://data.cdc.gov/api/views/qvzb-qs6p and accessed on January 2, 2025.^2^ We assume that 15B provides protection against 15C for all vaccines that include 15B due to rapid switching between these serotypes due to a point mutation. High-quality, population-based active surveillance for IPD in the United States currently covers a population of 35 million individuals from parts of 10 states. Reporting of IPD data lag by several years.

^b^Not for use in infant immunization program.

After a new PCV is introduced, there is a risk that colonization with serotypes not targeted by the PCV could increase in frequency among healthy people and might more frequently cause disease [4, 9]. This serotype replacement occurs because pneumococci compete to colonize the nasopharynx, so when colonization with vaccine-targeted serotypes decreases, other serotypes may thrive and colonize the empty ecologic niche. This phenomenon was clearly observed after PCV7 was introduced in children and when recommendations switched from PCV7 to either PCV10 or PCV13. For instance, in high-income countries, the near elimination of IPD caused by PCV7 serotypes among children and adults was partially offset by an increase in IPD caused by non-PCV7 serotypes [2]. There are still >80 serotypes not included in the PCVs being used in children, so we expect that there still will be serotype replacement among healthy carriers. The extent to which these emerging serotypes cause disease will depend on their pathogenicity and prevalence among healthy carriers. Likewise, if there is a switch to a PCV of lower valency, the serotypes no longer included in the vaccine may reemerge as a cause of disease, as occurred in Belgium after a switch from PCV13 to PCV10 [16].

PCVs often do not completely eliminate colonization or disease due to all vaccine-targeted serotypes. Serotypes 19A and 19F have persisted at a low frequency [17], and serotype 4 has recently reemerged as an important cause of disease in high-risk populations (eg, indigenous populations and people experiencing homelessness) [18]. It is unclear if this ongoing transmission results from inadequate effectiveness against colonization in children or from unrecognized reservoirs for transmission among adults. The future dynamics of colonization and disease due to specific serotypes are challenging to predict given the differing immunogenicity of new PCVs, as well as the complex ecology and ability of pneumococcus to generate new genetic variants that can result in vaccine evasion [19].

Decisions about which PCV to offer to children and adults are complicated by which PCV an individual previously received and the serotype distribution in a particular setting. For instance, PCV21/V116 might provide the broadest protection for the general population of older adults, but PCV20 might be more impactful in certain risk groups in which particular serotypes (eg, serotype 4) are more important. These complexities add to the already challenging decisions for clinicians and policy makers about how an individual's age, comorbid conditions, and vaccine history influence which PCV should be administered.

Several sources of data can be used to assess new PCVs. However, these data might not always fully align, complicating the understanding of potential vaccine effects. Evaluating efficacy against clinical outcomes prior to licensure requires impractical, large-scale clinical trials. Thus, a correlate of protection derived from data on vaccine efficacy and immunogenicity from trials of earlier-generation PCVs is used for licensing newer PCVs [20]. Yet, the link between immunogenicity and effectiveness may vary by age, underlying conditions, geographic locations, and PCV formulation and will differ for carriage and disease.

It will be critical to continually reassess the effects of new PCVs on the serotype distribution and the disease burden among different age and risk groups. This will help to inform which PCV is best to use at any given time and place for adults and which serotypes should be included in PCVs under development. High-quality population-based active surveillance for IPD in the United States currently covers a population of 35 million individuals from parts of 10 states. However, reporting of these data lag by several years, and colonization surveys remain limited, compromising the ability to fully understand serotype patterns shortly after PCV switches. For decisions about which PCV to recommend or which serotypes should be included in future PCVs, we need timely, high-quality serotype-specific surveillance data for IPD, pneumococcal pneumonia, and nasopharyngeal colonization with pneumococcus.

In conclusion, patients and clinicians have more options than ever for preventing pneumococcal disease. Further analysis of surveillance data and continual monitoring of changes in the pneumococcal serotypes that are responsible for disease and colonization are needed to ensure that PCVs are used optimally and updated as needed.

## Notes

***Author contributions***. Conceptualization: D. T., D. M. W., E. D. S. Supervision: D. M. W., E. D. S. Validation: D. T., D. M. W., E. D. S. Writing–original draft: D. T., D. M. W., E. D. S. Writing–review and editing: D. T., D. M. W., E. D. S. All authors read and approved the final manuscript.

***Financial support***. There was no specific funding tied to this work.
