# Correction to: The Complex Landscape of Updated Pneumococcal Conjugate Vaccines

**DOI:** 10.1093/ofid/ofaf265

**Published:** 2025-05-07

This is a correction to: Deus Thindwa, Eugene D Shapiro, Daniel M Weinberger, The Complex Landscape of Updated Pneumococcal Conjugate Vaccines, *Open Forum Infectious Diseases*, Volume 12, Issue 2, February 2025, https://doi.org/10.1093/ofid/ofaf050

The following changes have been made to the originally published manuscript. Table 1 has been emended and should read:

**Table 1.** Pneumococcal serotypes targeted by various pneumococcal conjugate vaccines (PCVs), including vaccines still under clinical investigations, and the proportion of invasive pneumococcal disease (IPD) preventable by each PCV

	Pneumococcal Serotype																																			PCV Coverage^a^	
PCV	4	6B	9V	14	18C	19F	23F	1	5	7F	3	6A	19A	22F	33F	8	10A	11A	12F	15B	2	9N	17F	20B	15A	16F	20A	23A	23B	24F	31	35B	15C	7C	6C	<5 years (%)	50+ years (%)
PCV7	x	x	x	x	x	x	x																													11.8	9.1
PCV10 (GSK)	x	x	x	x	x	x	x	x	x	x																										11.8	9.4
PCV13	x	x	x	x	x	x	x	x	x	x	x	x	x																							28.6	27.5
PCV15	x	x	x	x	x	x	x	x	x	x	x	x	x	x	x																					43.7	39.4
PCV20	x	x	x	x	x	x	x	x	x	x	x	x	x	x	x	x	x	x	x	x																53.8	50.6
SP0202 (21-valent)	x	x	x	x	x	x	x	x	x	x	x	x	x	x	x	x	x	x	x	x		x														53.8	57.4
MAPS24	x	x	x	x	x	x	x	x	x	x	x	x	x	x	x	x	x	x	x	x	x	x	x	x												54.6	63.3
VAX24	x	x	x	x	x	x	x	x	x	x	x	x	x	x	x	x	x	x	x	x	x	x	x	x												54.6	63.3
PCV25 (Inventprise)	x	x	x	x	x	x	x	x	x	x	x	x	x	x	x	x	x		x	x		x			x	x				x		x			x	77.3	73.1
VAX31	x	x	x	x	x	x	x	x	x	x	x	x	x	x	x	x	x	x	x	x	x	x	x	x	x	x		x	x		x	x		x		79.8	90.9
Pneumosil^®^ (10-valent)		x	x	x		x	x	x	x	x		x	x																							14.3	6.3
V116										x	x	x	x	x	x	x	x	x	x			x	x		x	x	x	x	x	x	x	x	x			^b^	81.4

^a^PCV coverage – proportion of serotyped invasive pneumococcal disease (IPD) targeted by each PCV based on 2022 IPD data from the United States Centers for Disease Control and Prevention Active Bacterial Core (ABC) surveillance project. PCV coverage data were obtained from https://data.cdc.gov/api/views/qvzb-qs6p and accessed on January 2, 2025.^2^ We assume that 15B provides protection against 15C for all vaccines that include 15B due to rapid switching between these serotypes due to a point mutation. High-quality, population-based active surveillance for IPD in the United States currently covers a population of 35 million individuals from parts of 10 states. Reporting of IPD data lag by several years.

^b^Not for use in infant immunization program.

instead of:

**Table 1.** Pneumococcal Serotypes Targeted by Various PCVs, Including Vaccines Still Under Clinical Investigations, and the Proportion of IPD Preventable by Each PCV

	Pneumococcal Serotype																																			PCV Coverage,^a^ %	
PCV	4	6B	9V	14	18C	19F	23F	1	5	7F	3	6A	19A	22F	33F	8	10A	11A	12F	15B	2	9N	17F	20B	15A	16F	20A	23A	23B	24F	31	35B	15C	7C	6C	<5 y	≥50 y
PCV7																																				11.8	9.1
PCV10 (GSK)																																				11.8	9.4
PCV13																																				28.6	27.5
PCV15																																				43.7	39.4
PCV20																																				53.8	50.6
SP0202 (21-valent)																																				53.8	57.4
MAPS24																																				54.6	63.3
VAX24																																				54.6	63.3
PCV25 (Inventprise)																																				77.3	73.1
VAX31																																				79.8	90.9
Pneumosil (10-valent)																																				14.3	6.3
V116																																				…^b^	81.4

Abbreviations: IPD, invasive pneumococcal disease; PCV, pneumococcal conjugate vaccine.

^a^PCV coverage: proportion of serotyped IPD targeted by each PCV based on 2022 IPD data from the US Centers for Disease Control and Prevention Active Bacterial Core surveillance project. PCV coverage data were obtained from https://data.cdc.gov/api/views/qvzb-qs6p (accessed 2 January 2025) [2]. We assume that 15B provides protection against 15C for all vaccines that include 15B due to rapid switching between these serotypes attributed to a point mutation. High-quality population-based active surveillance for IPD in the United States currently covers a population of 35 million individuals from parts of 10 states. Reporting of IPD data lags by several years.

^b^Not for use in infant immunization program.

The emendation has been made to the article.

